# Mitigation Effects of a Novel Herbal Medicine, Hepad, on Neuroinflammation, Neuroapoptosis, and Neuro-Oxidation

**DOI:** 10.3390/molecules23112920

**Published:** 2018-11-08

**Authors:** Da Hye Song, Gyeong-Ji Kim, Kwon Jai Lee, Jae Soo Shin, Dong-Hee Kim, Byung-Jun Park, Jeung Hee An

**Affiliations:** 1 Department of Food Science and Technology, Seoul National University of Science & Technology, Seoul 01811, Korea; sdh5740@naver.com; 2 Division of Food Bioscience, Konkuk University, Chungju 27478, Korea; kgj8495g@gmail.com; 3 Department of Biomedical Engineering, Sogang University, Seoul 04170, Korea; 4 Department of Advanced Materials Engineering, Daejeon University, Daejeon 34520, Korea; jmul@ssu.ac.kr (K.J.L); jsshin@dju.kr (J.S.S.); 5 Department of Pathology, College of Oriental Medicine, Daejeon University, Daejeon 34520, Korea; dhkim@dju.ac.kr

**Keywords:** Parkinson’s disease, 1-methyl-4-phenyl-1,2,3,6-tetrahydropyridine, Hepad, Mitigation effect, SH-SY5Y cells, Substantia nigra

## Abstract

Parkinson’s disease (PD), a common adult-onset neurodegenerative disorder with complex pathological mechanisms, is characterized by the degeneration of dopaminergic nigrostriatal neurons. The present study demonstrated that the herbal medicines Hepad 1 and 2 protected against 1-methyl-4-phenyl-1,2,3,6-tetrahydropyridine (MPTP)-induced dopaminergic neurotoxicity in C57BL/6 mice and SH-SY5Y cells. Hepad 1 and 2 remarkably alleviated the enhanced expression of pro-inflammatory cytokines (tumor necrosis factor-α, interleukin-6, inducible nitric oxide synthase, cyclooxygenase-2, macrophage-1, and phosphorylated iκB-α) and apoptotic signals (Bcl-2-associated X protein, caspase-3, and poly [ADP-ribose] polymerase-1). Additionally, Hepad reduced MPTP-induced oxidative damage by increasing the expression of anti-oxidant defense enzymes (superoxide dismutase and glutathione S-transferase) and downregulating the levels of nicotinamide adenine dinucleotide phosphate oxidase 4. This study also showed that the neuroprotective effects of Hepad include anti-inflammatory, anti-apoptotic, and anti-oxidative properties, in addition to activation of the protein kinase B, extracellular-signal-regulated kinase, and c-Jun N-terminal kinase signaling pathways. Furthermore, oral administration of Hepad 1 and 2 attenuated the death of tyrosine hydroxylase-positive substantia nigra neurons that was induced by 20 mg/kg MPTP. Therefore, our results suggest that Hepad 1 and 2 are useful for treating PD and other disorders associated with neuro-inflammatory, neuro-apoptotic, and neuro-oxidative damage.

## 1. Introduction

Parkinson’s disease (PD) is caused by a deficiency of the neurotransmitter dopamine at the nerve terminals of nigrostriatal dopaminergic neurons in the striatum owing to the selective loss of dopaminergic neurons in the substantia nigra pars compacta (SNpc) [1]. In addition, PD is characterized by various types of motor dysfunction, such as bradykinesia, rigidity of the limbs, and shuffling gait [2]. Potential mechanisms underlying the pathogenesis of PD include disturbances in intracellular calcium homeostasis, the presence of exogenous and endogenous toxins, mitochondrial dysfunction, death of nigrostriatal dopaminergic neurons, oxidative stress, and cytotoxicity of reactive oxygen species (ROS) [1,3]. These factors are likely part of a complex network that leads to the death of dopaminergic neurons in PD [4].

Treatment with 1-methyl-4-phenylpyridinium (MPP+) induces a severe parkinsonian-like syndrome and a significant reduction in dopaminergic cells by selectively inhibiting complex I in the mitochondrial electron transport chain [5,6]. Neurodegenerative processes are typically characterized by a long-lasting course of neuronal death [1]. Apoptosis is a form of programmed cell death that is accompanied by morphological changes, including cell shrinkage, nuclear condensation, and DNA degradation [1,7]. The apoptotic process is caused by a cascade of events, in which a family of cysteine proteases (caspases) mediates the cleavage of multiple cellular substrates [1,7]. Apoptotic death also alters the expression of multiple genes, many of which are *oncogenes*; some of these *oncogenes* enhance the apoptotic process (e.g., *Bcl2*-associated X [*Bax*] and B-cell lymphoma [*Bcl*]-x), whereas others serve to inhibit the process (*Bcl-2* and *Bcl-xL*) [8]. Moreover, the swelling and rupture of necrotic cells provoke an inflammatory response [1]. Neuroinflammation contributes to neurodegeneration and is thought to be primarily associated with overactive glial cells in the brains of patients with PD [4]. Microglial cells, which constitute resident macrophages in the brain, synthesize inflammatory factors, such as *cyclooxygenase-2* (*COX-2*), *tumor necrosis factor-alpha* (*TNF-α*), and *interleukin-6* (*IL-6*), as well as free radicals such as *nitric oxide* (*NO*) and superoxide [3,9,10,11,12,13,14]. Additionally, during neuroinflammation, it is known that NO and prostaglandin 2 production are mainly mediated by *inducible nitric oxide synthase* (*iNOS*) and *COX-2*, respectively [14]. These inflammatory factors are responsible for neuroglia-mediated neuroinflammation and neurotoxicity [15].

Currently, no existing drugs are capable of inhibiting or delaying the progression of PD [2]. Therefore, further studies are needed to identify compounds that can modulate the disease process and prevent the progression of symptoms in affected patients. Our research has focused on herbal medicines for the treatment of PD. In previous studies, herbal medicines, such as Hepad, which is composed of *Atractylodis Rhizoma*, *Cnidii Rhizoma*, *Paeonia japonica*, *Poria cocos Wolf*, *Uncariae Ramulus et Uncus*, and *Zizyphi Semen*, have been found to inhibit cell apoptosis by reducing activation of the caspase-9/caspase-3 pathway [16]. Regarding the biological effects of Hepad’s components, *Atractylodis Rhizoma Alba* has been found to exhibit anti-oxidative, gastroprotective, and anti-inflammatory activity [17]. *Cnidii Rhizoma* has been reported to exhibit anti-tumor activity and anti-angiogenic activity in an in vivo model [18]. Furthermore, paeoniflorin, a main constituent of *Paeonia japonica*, has been shown to prevent inflammation and stomachache. *Paeonia japonica* may also enhance immune function by modulating in vivo pro-inflammatory cytokines and *nitric oxide* (*NO*) production, as well as the expression of *iNOS* and *COX-2* [19]. Moreover, *Uncariae Ramulus et Uncus* has been shown to ameliorate learning and memory deficits by exerting neuroprotective effects on the central acetylcholine system [20]. However, the complete mechanisms underlying the beneficial effects of Hepad, including its anti-apoptotic, anti-inflammatory, and anti-oxidative activity, as well as its ability to regulate kinase signaling, have not yet been elucidated. Therefore, the present study aimed to evaluate the beneficial effects of Hepad in cell and animal models of PD.

## 2. Results

### 2.1. Hepad Ameliorates the 1-methyl-4-phenyl-1,2,3,6-tetrahydropyridine Hydrochloride (MPTP)-Induced Reduction in Neuronal Cell Viability

**Figure 1:**
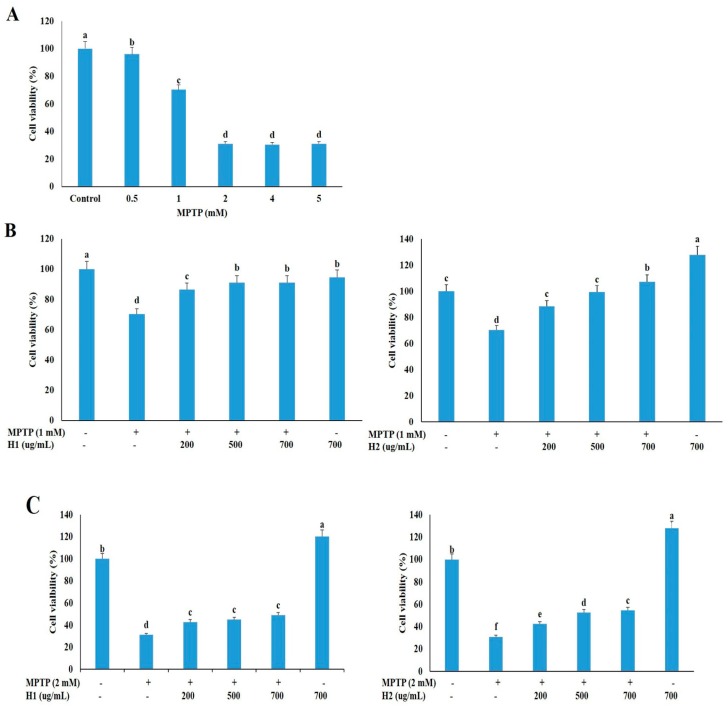
Hepad 1 (H1) and Hepad 2 (H2) inhibit 1-methyl-4-phenyl-1,2,3,6-tetrahydropyridine (MPTP)-induced cell death in SH-SY5Y cells. (**A**) SH-SY5Y cells (2 × 10^6^) were incubated in the absence or presence of MPTP for 24 h. SH-SY5Y cells were pre-treated with 1 mM (**B**) and 2 mM (**C**) MPTP for 4 h, and then incubated with 200, 500, and 700 µg/mL of H1 and H2 (S5) for 24 h. Subsequently, the survival rate was measured with a 3-(4,5-dimethylthiazol-2-yl)-2,5-diphenyltetrazolium bromide assay. The data are expressed as the relative ratio to the absorbance of the untreated cells, which was set at 100%, and are reported as the mean ± standard deviation of three independent experiments. Level of statistical significance for a, b, c, d, e and f is *p* < 0.05 (Duncan’s multiple range test).

We treated SH-SY5Y neuronal cells with various concentrations of MPTP (0, 0.5, 1, 2, 4, and 5 mM) for 24 h, and then measured the cell viability with the 3-(4,5-dimethylthiazol-2-yl)-2,5-diphenyltetrazolium bromide (MTT) assay to determine an appropriate concentration for subsequent studies. The cell viability was dramatically reduced in cells treated with 0.5 mM to 5 mM MPTP compared to untreated cells (Figure 1A); thus, 1 and 2 mM MPTP, which caused significant cell death (29.05% and 68.89%, respectively), were selected to investigate the protective effects of Hepad 1 (H1) and Hepad 2 (H2) in subsequent experiments. As shown in Figure 1B, cell viabilities increased by 86.5%, 91.2%, and 91.2% after treatment with 200, 500, and 700 μg/mL H1, respectively, compared with cells treated with 1 mM MPTP alone. Treatment with H2 (200, 500, and 700 μg/mL) also alleviated the MPTP-induced cell toxicity in a concentration-dependent manner. In particular, treatment with 700 μg/mL H2 significantly attenuated (57.6%) the cell toxicity of MPTP (Figure 1B). In 2-mM MPTP-intoxicated cells, treatment with 200, 500, and 700 μg/mL H1 significantly increased the cell viability by 42.85%, 44.96%, and 48.97%, respectively (Figure 1C). Moreover, treatment with H2 significantly rescued the reduced cell viability in 2-mM MPTP-intoxicated cells in a concentration-dependent manner; cells treated with 700 μg/mL H2 showed the greatest viability (48.97%) (Figure 1C). Collectively, these results demonstrated that H1 and H2 can rescue neuronal cell viability during treatment with a toxic compound. Based on these results, 2 mM MPTP was used in subsequent experiments to examine the mechanisms of PD.

### 2.2. Hepad Attenuates MPTP-Induced Inflammation

**Figure 2:**
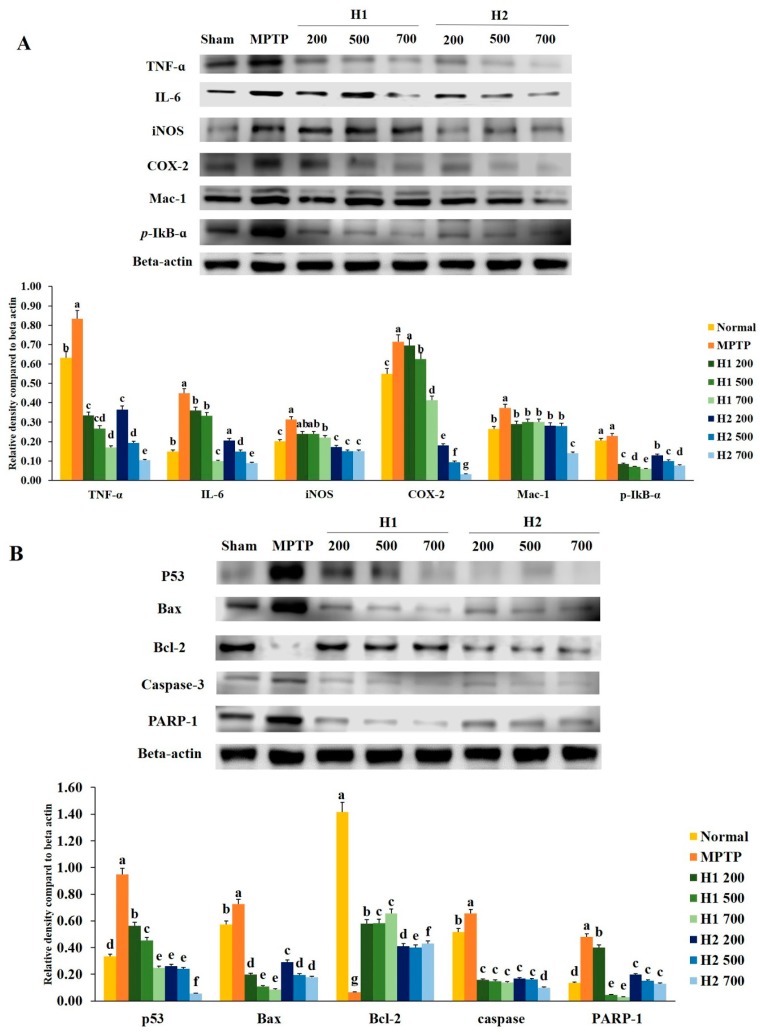
Alleviation of increased expression of (**A**) inflammation-related proteins and (**B**) apoptosis-related proteins in 1-methyl-4-phenyl-1,2,3,6-tetrahydropyridine (MPTP)-intoxicated SH-SY5Y cells, following treatment with Hepad 1 (H1) and Hepad 2 (H2) (S5). Results are expressed as the mean ± the standard deviation. Level of statistical significance for a, b, c, d, e, f and g is *p* < 0.05 (Duncan’s multiple range test).

To investigate the anti-inflammatory effects of H1 and H2, we examined whether H1 and H2 would affect the activation of *TNF-α*, *IL-6*, *iNOS*, *COX-2*, *macrophage-1* (*Mac-1*), and *phosphorylated IκB-α* (*p-IκB-α*). As shown in Figure 2A, a significant elevation in the *TNF-α* protein expression level was found in MPTP-intoxicated cells compared with the expression level in control cells; this elevation was significantly attenuated by treatment with 200, 500, and 700 µg/mL H1. In addition, treatment with H2 (200, 500, and 700 μg/mL) significantly alleviated the elevated expression of *TNF-α* in a concentration-dependent manner (Figure 2A).

The level of *IL-6* was significantly elevated in MPTP-intoxicated cells relative to the level in control cells, but the *IL-6* level was significantly reduced after treatment with 200, 500, and 700 µg/mL H1 (Figure 2A). Moreover, treatment with H2 reduced the expression levels of *IL-6* in a concentration-dependent manner (Figure 2A). Therefore, Hepad treatment suppresses *IL-6* expression, an indicator of inflammation. The results demonstrate the anti-inflammatory effects of Hepad in MPTP-intoxicated cells. Moreover, MPTP significantly elevated the level of *iNOS* relative to the level in control cells; this secretion was blocked by treatment with either H1 or H2 (Figure 2A).

The expression of *COX-2* was significantly elevated in MPTP-treated vs. control SH-SY5Y cells; this enhanced secretion was repressed by treatment with 500, and 700 µg/mL H1 (1.1-, and 1.7-fold, respectively) (Figure 2A). Furthermore, compared to the expression of *COX-2* protein in cells treated with MPTP alone, the *COX-2* expression levels in MPTP-intoxicated cells after treatment with 200, 500, and 700 µg/mL H2 were significantly reduced by 4.0-, 7.6-, and 22.6-fold, respectively (Figure 2A).

The expression of *Mac-1* protein in 2-mM MPTP-intoxicated cells was significantly elevated (by 0.8-fold) compared with the expression in control cells; this elevation was attenuated by treatment with 700 µg/mL H2 (2.7-fold) (Figure 2A).

The expression of *p-IκB-α* protein was higher in MPTP-intoxicated cells (Figure 2A) than it was in control cells. The MPTP-induced upregulation of *p-IκB-α* was reversed by treatment with either H1 or H2; treatment with 700 µg/mL H1 or H2 demonstrated a maximum reversal effect (Figure 2A). Here, Western blotting analyses demonstrated that inflammatory response proteins play a pivotal role in MPTP-induced inflammation. Our results also indicated that the expression levels of inflammation-related proteins, such as *IL-6, COX-2,* and *Mac-1*, were reduced after Hepad treatment. Thus, our results suggest that Hepad may mitigate PD-associated pathology.

### 2.3. Effects of Hepad on Pro-Apoptotic and Anti-Apoptotic Protein Expression in MPTP-Intoxicated SH-SY5Y Cells

To evaluate the effects of Hepad treatment on anti-apoptotic signaling mechanisms, we investigated whether Hepad alters the activation of pro-apoptotic and anti-apoptotic proteins.

Figure 2B shows that *p53* protein expression was significantly elevated after MPTP induction compared with the expression in untreated cells; the *p53* protein expression was significantly reduced after treatment with H1 and H2 compared with its expression after MPTP induction. Furthermore, as shown in Figure 2B, the level of *Bax* was robustly elevated in MPTP-intoxicated cells vs. control cells; treatment with H1 or H2 significantly suppressed the enhanced expression of *Bax* in SH-SY5Y cells. In contrast, expression of the anti-apoptotic protein *Bcl-2* was significantly inhibited in MPTP-intoxicated cells relative to the expression in control cells, but was significantly elevated in cells treated with 200, 500, and 700 µg/mL of H1 or H2 (Figure 2B).

We observed that MPTP intoxication induced cleavage of *caspase-3*, but this cleavage was significantly inhibited by treatment with 200, 500, and 700 µg/mL of H1 or H2. The expression levels of *poly (ADP-ribose) polymerase-1* (*PARP-1*) protein were significantly lower in cells treated with 200, 500, and 700 µg/mL H1 than they were in MPTP-intoxicated cells (Figure 2B). Moreover, treatment with H2 (200, 500, and 700 µg/mL) significantly reduced the expression levels of *PARP-1* protein in a concentration-dependent manner (by 2.44-, 3.19-, and 3.68-fold, respectively) (Figure 2B). Therefore, Hepad might serve to alleviate PD by suppressing activation of the apoptosis signaling pathway.

### 2.4. Hepad Suppresses MPTP-Induced Oxidative Stress

**Figure 3:**
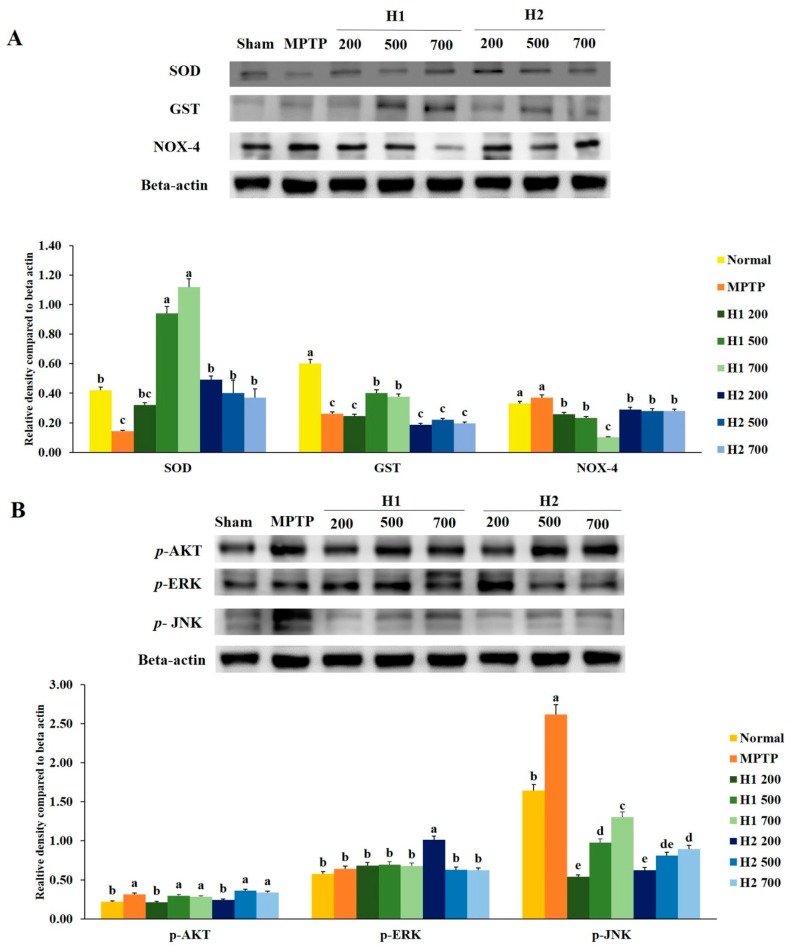
Mitigation effects of Hepad 1 (H1) and Hepad 2 (H2) (S5) on the expression of anti-oxidant enzymes in 1-methyl-4-phenyl-1,2,3,6-tetrahydropyridine (MPTP)-intoxicated SH-SY5Y cells (**A**). Expression levels of protein kinase B (AKT), c-Jun N-terminal kinase (JNK), and extracellular signal-regulated kinase (ERK) in MPTP-treated SH-SY5Y cells (**B**). Results are expressed as the mean ± the standard deviation. Level of statistical significance for a, b, c, d, and e is *p* < 0.05 (Duncan’s multiple range test).

The expression of *superoxide dismutase* (*SOD*) protein was significantly reduced in MPTP-intoxicated cells compared with the expression in control cells. Notably, treatment with H1 increased the expression levels of *SOD* protein in a concentration-dependent manner (Figure 3A). Furthermore, the expression level of *SOD* was significantly higher in H2-treated cells than it was in MPTP-intoxicated cells; however, no significant differences were found among cells treated with various concentrations of H2 (Figure 3A). Similarly, compared to the *glutathione S-transferase* (*GST*) expression levels in control cells, the expression levels of *GST* in MPTP-intoxicated cells were significantly reduced. The expression levels of *GST* protein were elevated by treatment with 500, 700 µg/mL H1 relative to the levels in MPTP-intoxicated cells. But these differences were not statistically significant. Expression of *nicotinamide adenine dinucleotide phosphate oxidase 4* (*NOX-4*) was slightly, but not significantly, elevated in MPTP-intoxicated vs. control cells; this activation was significantly inhibited by treatment with 200, 500, and 700 µg/mL H1 (1.43-, 1.6-, and 3.7-fold, respectively). Notably, treatment with H2 reduced the expression levels of *NOX-4* (Figure 3A).

### 2.5. Hepad Attenuates the Elevation of Phosphorylated Protein Kinase B (p-AKT) and Mitogen-Activated Protein Kinase (MAPK) in MPTP-Intoxicated SH-SY5Y Cells

We assessed the effects of the Hepad extract on *p-AKT*, *phosphorylated extracellular-signal-regulated kinase* (*p*-*ERK*), and *phosphorylated c-Jun N-terminal kinase* (*p*-*JNK*) in MPTP-stimulated SH-SY5Y cells. Treatment with 200 µg/mL H1 or H2, but not 500 and 700 µg/mL H1 or H2, significantly suppressed the enhanced expression of *p*-*AKT* in MPTP-intoxicated cells (Figure 3B).

Stimulation with MPTP slightly, but not significantly, elevated the level of *p*-*ERK* compared to the level in control cells. However, subsequent treatment with 200, 500, and 700 µg/mL H1 or 500 and 700 µg/mL H2 slightly inhibited the activation of *p*-*ERK* in MPTP-intoxicated cells; the levels of *p*-*ERK* decreased by 1.1-, 1.2-, and 1.3-fold after treatment with H1 and 1.6-, 1.0-, and 1.0-fold after treatment with H2, respectively (Figure 3B).

The *p*-*JNK* signaling pathway has been implicated in the onset of apoptosis following numerous types of stress, such as nerve growth factor withdrawal, excitotoxic stress, and oxidative stress [21,22]. Consistent with these prior observations, MPTP-intoxicated cells showed upregulated protein levels of *p*-*JNK* compared with the level in control cells; this indicated the activation of *p*-*JNK* in MPTP-intoxicated SH-SY5Y cells. Notably, subsequent treatment with H1 or H2 (200, 500, and 700 µg/mL) significantly attenuated the MPTP-induced elevation of *p*-*JNK* in SH-SY5Y cells (Figure 3B).

### 2.6. Hematoxylin and Eosin (H&E) Staining and Immunohistochemical Detection of Tyrosine Hydroxylase (TH) in the substantia nigra (SN) of Mice

**Figure 4:**
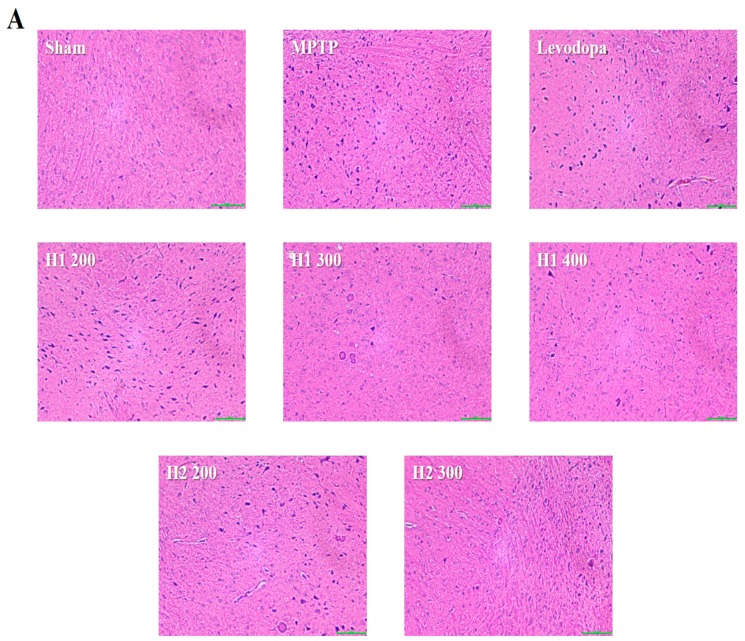

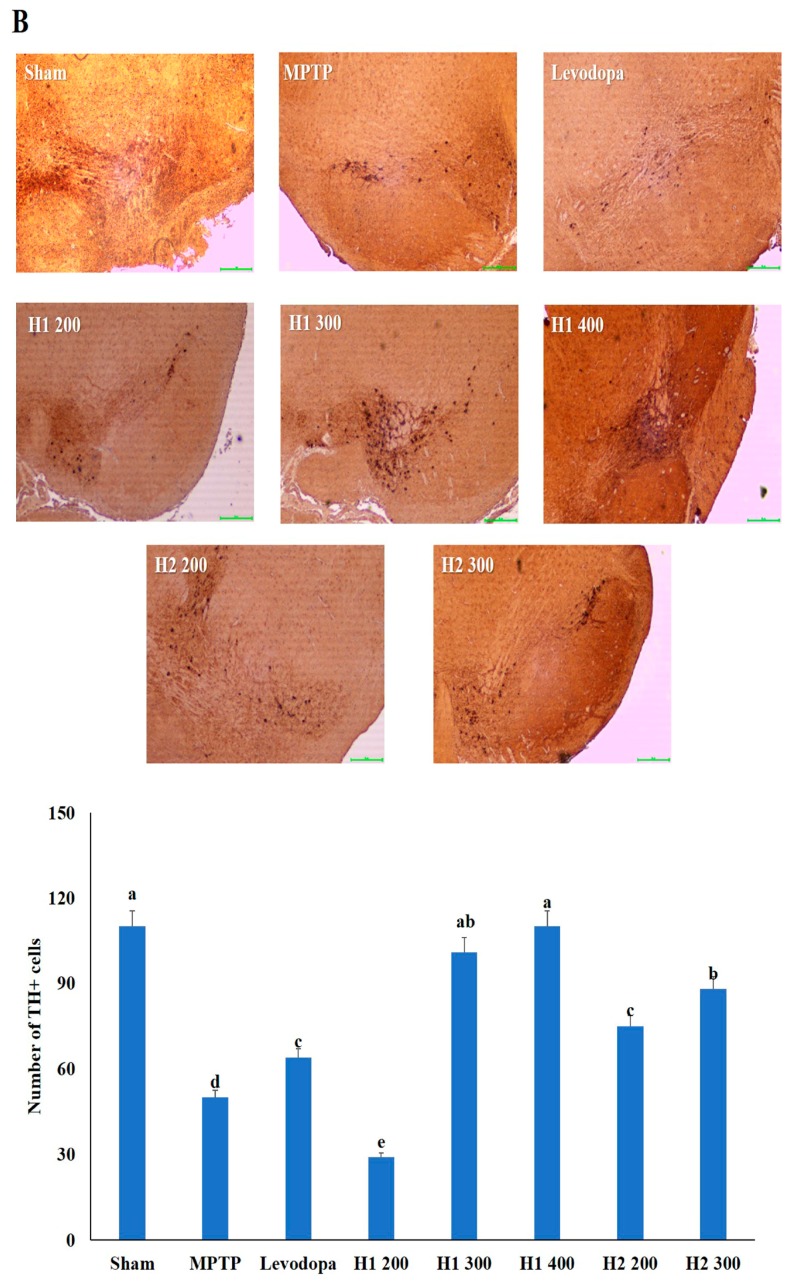
Representative photomicrographs (40× magnification) of (**A**) hematoxylin and eosin-stained brain sections and (**B**) immunohistochemical staining of tyrosine hydroxylase -positive (TH+) neurons (scale bar, 100 µm). Results are expressed as the mean ± the standard deviation. Level of statistical significance for a, b, c, d, and e is *p* < 0.05 (Duncan’s multiple range test).

Lewy bodies were observed as spherical bodies, each with a dense core surrounded by a halo, in MPTP-intoxicated mice compared with the sham mice (Figure 4). Brain specimens from MPTP-intoxicated mice showed dopaminergic neuronal damage with the loss of the multipolar shape and distorted nuclei (Figure 4). Notably, multipolar neurons with nucleoli and basophilic granular cytoplasm were observed in the SNpc in H1- and H2-treated mice; moreover, the neuronal damage was improved after treatment with H1 or H2 in a concentration-dependent manner (Figure 4). Our results further demonstrated that H1 and H2 exhibited better mitigation effects than did the positive control, levodopa.

The number of TH-positive cells in the MPTP-intoxicated group was reduced by more than 2-fold compared with the number in the sham group (Figure 4). Numbers of TH-positive cells were significantly elevated in the groups treated with levodopa, H1 (300, and 400 mg/kg), or H2 (200 and 300 mg/kg) compared with the MPTP-intoxicated group (2.0-fold, and 2.2-fold after treatment with H1; 1.5- and 1.76-fold after treatment with H2, respectively) (Figure 4).

### 2.7. Mitigation Effects of Hepad on MPTP-Induced Inflammatory Responses

**Figure 5:**
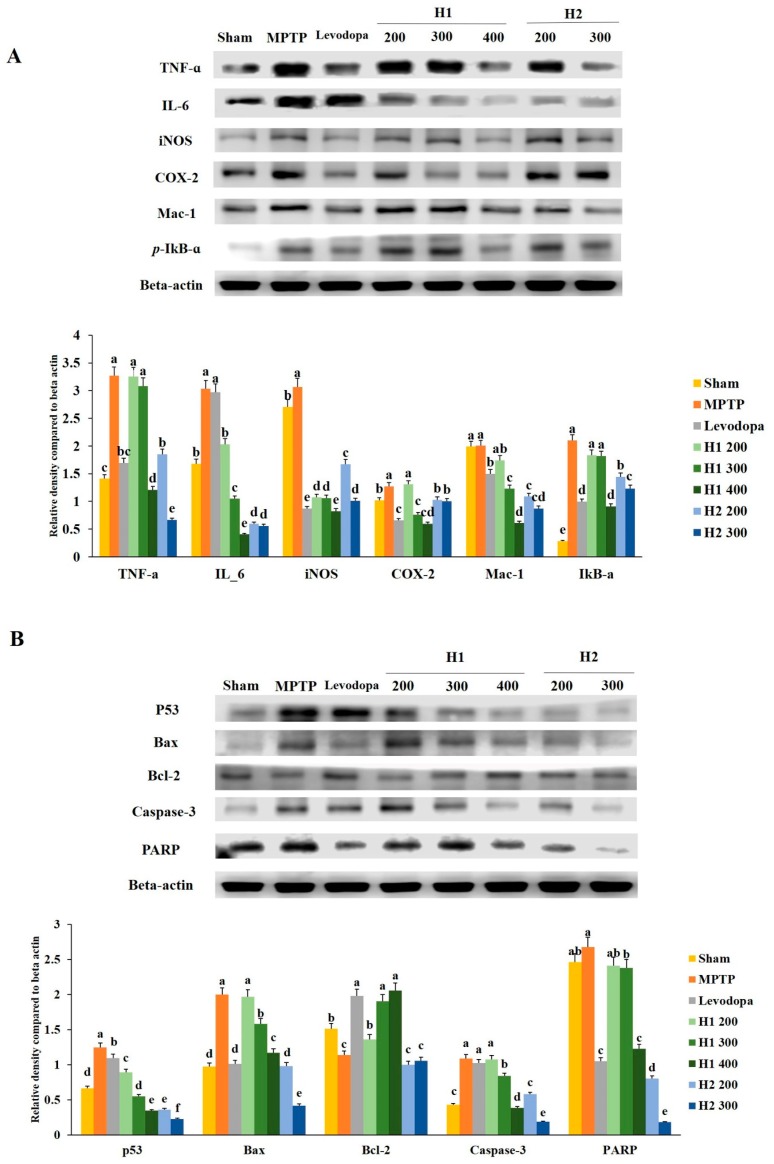
Effects of Hepad 1 (H1) and 2 (H2) (S5) on (**A**) inflammation-related responses and (**B**) apoptotic signaling cascades in 1-methyl-4-phenyl-1,2,3,6-tetrahydropyridine (MPTP)-treated mice. Protein extracts from brain tissues in each of the different groups were subjected to Western blotting analyses. Results are expressed as the mean ± the standard deviation. Level of statistical significance for a, b, c, d, e, and f is *p* < 0.05 (Duncan’s multiple range test).

Inflammatory responses were generally triggered in the MPTP model. The expression of *TNF*-α was significantly higher in the MPTP-intoxicated group than it was in the vehicle-treated group. The expression levels of *TNF*-α protein were significantly downregulated in the groups treated with high doses of H1 or H2 compared with the level in the MPTP-intoxicated group (2.7-fold after treatment with H1 400 mg/kg and 4.9-fold after treatment with H2 300 mg/kg) (Figure 5A).

Additionally, the expression of *IL-6* protein, which is an inflammation-related protein, was significantly elevated in MPTP-intoxicated vs. sham mice; however, this increased expression was significantly attenuated in a dose-dependent manner by treatment with H1 (200, 300, and 400 mg/kg; 1.5-, 2.9-, and 7.5-fold, respectively) or H2 (200 and 300 mg/kg; 5.1- and 5.4-fold, respectively) (Figure 5A).

The *iNOS* protein expression level was elevated by 0.44-fold in the MPTP-intoxicated group compared with the level in the sham group; however, this elevated expression was significantly inhibited by treatment with H1 (2.9-, 2.9-, and 3.7-fold after treatment with 200, 300, and 400 mg/kg H1, respectively) or H2 (1.8- and 3.0-fold after treatment with 200 and 300 mg/kg H2, respectively) (Figure 5A). Furthermore, compared to the *COX-2* level in the sham group, the MPTP-intoxicated group exhibited significantly elevated *COX-2* activity, while treatment with 300 and 400 mg/kg H1 and 200 and 300 mg/kg H2 significantly attenuated this elevated activity (Figure 5A).

The *Mac-1* protein levels were lower in the groups treated with 200, 300, and 400 mg/kg H1 (1.2-, 1.6-, and 3.3-fold, respectively) than they were in the MPTP-intoxicated group; these reductions were dose-dependent (Figure 5A). In addition, *Mac-1* expression was reduced in the groups treated with 200 and 300 mg/kg H2 (1.8- and 2.3-fold, respectively) compared with the expression in the MPTP-intoxicated group (Figure 5A).

We noted that the *p*-*IκB*-α levels were dramatically elevated after MPTP intoxication and that levodopa slightly restored the *p*-*IκB*-α level to its baseline value (Figure 5A). Further, the *p*-*IκB*-α levels were downregulated in the groups treated with 200, 300, and 400 mg/kg H1 compared with the levels in the MPTP-intoxicated group. Treatment with 200 and 300 mg/kg H2 significantly reduced the *p*-*IκB*-α expression levels compared with the levels in the MPTP-intoxicated group; this downregulation was dose-dependent (Figure 5A). Taken together, our results indicate that H1 and H2 downregulated the elevated expression levels of a variety of inflammation-related proteins, such as *TNF*-α, *IL-6*, *iNOS*, *Mac-1*, and *p*-*IκB*-α, in MPTP-intoxicated mice (Figure 5A). Therefore, these results demonstrate the ability of Hepad to mitigate neuroinflammation in the MPTP-induced mouse model of PD.

### 2.8. Hepad Affects the Activation of Pro-Apoptotic and Anti-Apoptotic Proteins

To examine the effects of Hepad on MPTP-induced cell apoptosis, we investigated several proteins involved in the apoptotic process. The expression of *p53* protein was significantly upregulated by 1.9-fold in MPTP-intoxicated mice compared with the expression level in sham mice (Figure 5B). However, the upregulation was significantly suppressed in mice treated with 200, 300, and 400 mg/kg H1 (1.4-, 2.3-, and 3.6-fold, respectively) or 200 and 300 mg/kg H2 (3.5- and 5.5-fold, respectively) (Figure 5B).

We observed that MPTP intoxication enhanced the expression levels of *Bax* protein compared to the levels in sham mice and that this enhancement was reduced by treatment with 200, 300, and 400 mg/kg H1 (1.0-, 1.3-, 1.7-fold, respectively), as well as 200 and 300 mg/kg H2 (2.0- and 4.8-fold, respectively) (Figure 5B). Additionally, the reduced *Bcl-2* expression that was observed in MPTP- intoxicated vs. sham mice was significantly reversed by treatment with 200, 300, and 400 mg/kg H1 (1.2-, 1.7-, and 1.8-fold, respectively), but not by treatment with 200 and 300 mg/kg H2; the H1 effects (300 and 400 mg/kg) were similar to the effects observed upon treatment with levodopa (positive control) (Figure 5B).

The expression levels of cleaved *caspase-3* in the H1 (300 and 400 mg/kg) and H2 (200 and 300 mg/kg)-treated groups were significantly reduced in a dose-dependent manner compared with the expression levels of cleaved *caspase-3* in the MPTP-intoxicated group (*p* < 0.05 for all) (Figure 5B). The expression of *PARP-1* protein was higher in the MPTP-intoxicated group than in the sham group. Treatment with H1 (200, 300, and 400 mg/kg) robustly blocked the MPTP-induced upregulation of *PARP-1* (1.1-, 1.2-, and 2.2-fold, respectively) (Figure 5B). In addition, treatment with H2 (200 and 300 mg/kg) significantly inhibited the MPTP-induced upregulation of *PARP-1* (Figure 5B). These results suggest that Hepad treatment effectively attenuates pro-apoptotic signals and elevates anti-apoptotic signals in brains exposed to MPTP intoxication.

### 2.9. Hepad Inhibits MPTP-Induced Oxidative Stress

**Figure 6:**
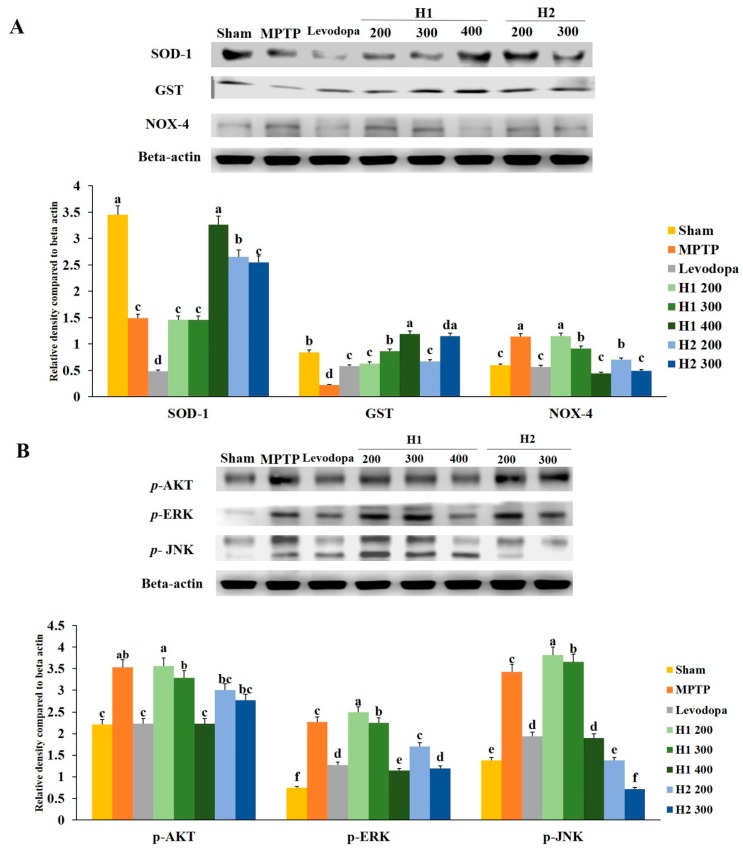
(**A**) Effects of Hepad 1 (H1) and Hepad 2 (H2) (S5) on the expression of anti-oxidant-related proteins in the brains of the Parkinson’s disease mouse model. Superoxide dismutase (SOD), glutathione S-transferase (GST), and nicotinamide adenine dinucleotide phosphate oxidase 4 (NOX-4) levels were assessed by Western blotting. (**B**) Effects of H1 and H2 (S5) on the expression of phosphorylated protein kinase B (*p*-AKT), phosphorylated extracellular signal-regulated kinase (*p*-ERK), and phosphorylated c-Jun N-terminal kinase (*p*-JNK) in the brains of the Parkinson’s disease mouse model. Results are expressed as the mean ± the standard deviation. Level of statistical significance for a, b, c, d, e, and f is *p* < 0.05 (Duncan’s multiple range test).

The expression level of *SOD* was significantly lower (2.3-fold) in the MPTP-intoxicated group than it was in the sham group; treatment with 400 mg/kg H1 and 200 mg/kg H2 significantly inhibited the MPTP-induced reductions in *SOD* (2.2- and 1.7-fold, respectively) (Figure 6A).

Similarly, the *GST* level in the MPTP-intoxicated group was reduced compared to the level in the sham group; treatment with 200, 300, and 400 mg/kg H1 (2.9-, 3.9-, and 5.4-fold, respectively) and 200 and 300 mg/kg H2 (3.0- and 5.2-fold, respectively) blocked the reduction effects of MPTP intoxication (Figure 6A). In addition, MPTP intoxication significantly upregulated the expression of *NOX-4* relative to the expression in the sham group; treatment with 300 and 400 mg/kg H1 (1.24- and 2.57-fold, respectively) and 200 and 300 mg/kg H2 (1.63- and 2.3-fold, respectively) significantly reduced the expression level of *NOX-4* relative to the MPTP-intoxicated group (Figure 6A).

### 2.10. Hepad Modulates the p-AKT and MAPK Signaling Pathways

We observed significantly elevated expression levels of *p*-*AKT*, *p*-*ERK*, and *p*-*JNK* proteins in the substantia nigra of MPTP-intoxicated vs. sham mice (Figure 6B). The MPTP-induced upregulation of *p*-*AKT* was attenuated by treatment with 200, 300, and 400 mg/kg H1 (1.0-, 1.1-, and 1.6-fold, respectively) (Figure 6B). Moreover, the upregulation of *p*-*AKT* was inhibited in groups treated with 200 and 300 mg/kg H2 (1.2- and 1.3-fold, respectively). Treatment with H1 (1.0 -, 1.0-, and 2.0-fold, respectively) or H2 (1.3- and 1.9-fold after H2 treatment, respectively) attenuated the elevated expression of the *p*-*ERK* protein. The MPTP-induced upregulation of *p*-*JNK* expression was also diminished by treatment with H1 or H2 (Figure 6B).

## 3. Discussion

The crucial characteristics of parkinsonism, including impaired motor function, decreased dopamine content, and reduced number of dopaminergic neurons (cell bodies and axonal terminals) in the nigrostriatal system, were replicated here in MPTP-intoxicated mice. In previous studies, we reported the apoptotic mechanisms by which Hepad exerts its protective effects in cell and animal PD models induced by exposure to 6-hydroxydopamine (6-OHDA) [16]. In the present study, we prepared H1 by mixing *Atractylodis Rhizoma*, *Cnidii Rhizoma*, *Paeonia japonica*, *Poria cocos Wolf*, *Uncariae Ramulus Et Uncus*, and *Zizyphi Semen* [16] and H2 by mixing *Paeonia japonica*, *Uncariae Ramulus et Uncus*, and *Machilus thunbergii*. To our knowledge, this is the first study to demonstrate that H1 and H2 exert their neuroprotective effects by regulating inflammation, anti-oxidant enzymes, and apoptosis in the SH-SY5Y cell and mouse PD models that are induced by MPTP intoxication.

Chronic inflammation is associated with a broad spectrum of age-related neurodegenerative diseases, including Alzheimer’s disease (AD), PD, amyotrophic lateral sclerosis, tauopathies, and macular degeneration [23]. In particular, the inflammatory responses, which are characterized by the activation of microglia and accumulation of inflammatory mediators (e.g., inflammatory cytokines and proteases) in the SN and striatum, are thought to be responsible for the progression of PD [24]. In the present study, treatment with 20 mg/kg MPTP produced PD signs in SH-SY5Y cell and mouse models, as indicated by significantly upregulated levels of inflammatory cytokines, such as *TNF*-α, *IL-6*, *iNOS*, *COX-2*, *Mac-1*, and *p*-*IκB*-α (*p* < 0.05). Knaryan et al. [25] showed results that were similar to ours. In addition, Feng et al. [26] found that the levels of *TNF*-α, *IL*-6, and *iNOS* increased in the MPTP-intoxicated group compared with the control group. Thus, inflammatory responses are upregulated in the PD model. Moreover, we assessed the mitigation effects of H1 and H2 on changes in pro-inflammatory cytokines and mediators in SH-SY5Y cell and mouse PD models. Interestingly, MPTP intoxication elevated the expression levels of *TNF*-α, *IL-6*, *iNOS*, *COX-2*, *Mac-1*, and *p*-*IκB*-α, but these elevated levels were markedly reduced by treatment with H1 or H2. Similarly, it has previously been revealed that *TNF*-α mRNA expression is significantly mitigated by melatonin treatment [27]. Yan et al. [28] found that clodronate liposome treatment reduced the mRNA expression of inflammatory cytokines, such as *TNF*-α, *IL*-1β, and *IL*-6, in the striatum and SNpc. Research has also shown that treatment with pioglitazone attenuates the activation of microglia and astrocytes in the striatum and SNpc in MPTP-intoxicated mice [29]. Moreover, *Poria cocos Wolf* mixture extracts elevate the expression of anti-inflammatory cytokines and reduce the levels of inflammatory cytokines [30]. Reduced levels of *iNOS*, *COX-2*, *p*-*IκB*-α, and *Mac-1* are known to regulate the anti-inflammatory responses to potentially toxic agents [31]. Furthermore, the ethanol extract of *Poria coccus*, which is regarded as a traditional herbal medicine for the treatment of inflammation, targets the inflammatory response of macrophages by inhibiting *iNOS*, *COX-2*, *IL-1β*, and *TNF-*α via the inactivation of the nuclear factor kappa B signaling pathway in RAW264.7 cells [32]. Therefore, our results support the anti-inflammatory potential of H1 and H2 in PD.

The molecular pathogenesis of PD is believed to be associated with mitochondrial dysfunction, oxidative stress, and activation of the apoptotic cascade [33]. It has been shown that the SNpc dopaminergic neurodegeneration that is associated with complex I deficiency occurs, at least partially, through activation of mitochondria-dependent apoptotic molecular pathways [34]. Activation of the apoptotic cascade may play a role in MPP+-induced cell death by altering mitochondrial membrane permeability and controlling the release of cytochrome C from mitochondria [33,35,36]. It has been shown that activated *caspase-9* and *caspase-3*, released by *cytochrome C*, are involved in MPP^+^-induced apoptosis [33,37,38,39]. Once activated, caspase-3 will induce nuclear DNA condensation and fragmentation and, ultimately, apoptosis [33].

Previously, Xia et al. [40] found that MPTP induced apoptotic cell death through *Bax* and caspase-3. Recently, it has also been reported that the expression level of *cytochrome C* is upregulated by MPTP intoxication [41]. Similarly, *PARP-1* is reportedly cleaved after MPTP treatment [42]. Indeed, our study showed that MPTP treatment significantly elevated the expression levels of pro-apoptotic proteins (*p53*, *Bax*, *caspase-3*, and *PARP-1*) and reduced the expression levels of anti-apoptotic signals (*Bcl-2*). One previous study showed that after apoptosis was induced by stimulation with 6-OHDA, *caspase-9* and *caspase-3* were cleaved, and then induced the downstream signaling of apoptosis [16]. In addition, the previous study indicated a protective effect of Hepad and showed that the expression levels of *caspase-9* and *caspase-3* decreased in a dose-dependent manner in the Hepad-treated group. Our present results are consistent with those of our previous study, in which H1 and H2 protected against MPTP-induced damage in SH-SY5Y cells. However, we only investigated the caspase family of proteins in our previous study; therefore, our present study investigated various apoptosis-related proteins, including *p53*, *Bax*, *Bcl-2*, and *PARP-1*. The present study found that the MPTP-induced damage could be reversed by treatment with H1 or H2. Another study indicated that talipexole (a dopamine agonist marketed as a treatment drug for PD) reduces MPTP-induced apoptotic signals, such as *Bax* and *p53*; moreover, it inhibits MPTP-induced cleavage of *caspase-3* and *PARP-1* [42]. In addition, bee venom increases the expression of anti-apoptotic Bcl-2 and decreases the levels of pro-apoptotic *Bax* and cleaved *PARP-1* [43]. Similarly, 6-OHDA-induced upregulation of *caspase-3* is reversed by treatment with *Uncariae Ramulus et Uncus* [44]. Thus, our results demonstrate that H1 and H2 may mitigate the expression of *Bax*/*Bcl-2* proteins in response to MPTP treatment and that they may regulate mitochondria-mediated downstream molecular events, including *caspase-3* activation and *PARP-1* proteolysis.

The impaired integrity of mitochondrial membranes not only destroys the transmembrane proton gradient and interrupts the synthesis of ATP, but also indirectly induces upregulation of ROS and the loss of enzyme activity; it may even trigger apoptosis [45]. Recently, oxidative stress has been recognized as an important pathological process in PD [46]. The degeneration of dopaminergic neurons induced by mitochondrial dysfunction and oxidative stress is an important feature of PD [46]. Moreover, it is well-established that oxidative stress plays a prominent role in the process of aging, making neurons more vulnerable to degeneration and the development of neurodegenerative disorders [47]. In previous studies, the production of ROS was observed after 6-OHDA treatment; in the present study, changes in the activity of anti-oxidant enzymes were detected after MPTP injection [16]. Therefore, the changes in anti-oxidant enzyme activity that we observed in the present study are consistent with the changes in ROS production that have been reported in previous studies. Interestingly, the current study showed the same pattern of *Bcl-2* expression as that reported in the study by Liu et al. [48]. Additionally, the levels of blood *SOD* and *GST* have been shown to decrease in MPTP-treated monkeys [49]. Notably, the expression levels of anti-oxidant enzymes were elevated by H1 and H2 treatment in the present study. An et al. [50] recently showed that treatment with *Gastrodia elata Blume* rescued dopaminergic neurons via the upregulation of *Bcl-2* activation in MPTP-intoxicated cells. Using the MPTP-intoxicated mouse model, Yue et al. [51] studied anti-oxidant enzyme activity and found that MPTP reduced the activity of *SOD*, whereas geranylgeranylacetone upregulated the anti-oxidant enzyme level. Furthermore, the expression level of *NOX-4*, which is associated with ROS production, was downregulated, along with the expression and activity of the anti-oxidant enzymes [52]. Xu et al. [53] also demonstrated that the *Machilus thunbergii* extract had anti-oxidant effects. These effects may be due to the ability of Hepad to directly scavenge ROS. Therefore, our results suggest that Hepad attenuates MPTP-induced dopaminergic neuronal injury.

To explore the signaling pathway involved in the neuroprotective effects of Hepad, the *p*-*AKT*, *p*-*ERK*, and *p*-*JNK* signaling pathways were investigated in the present study. The levels of *p*-*AKT*, *p*-*ERK*, and *p*-*JNK* were elevated in MPTP-intoxicated SH-SY5Y cell and mouse PD models. Notably, *p*-*AKT* levels markedly increased in the MPTP-intoxicated group; however, Hepad treatment alleviated the upregulation of *p*-*AKT* caused by MPTP. These results are consistent with those in previous studies, which showed that bee venom, amentoflavone, and curcumin reduced the *AKT*, *ERK*, and *JNK* protein expression levels in the MPTP-induced model [43,54,55]. These results indicate that Hepad mitigates the dopaminergic neuronal damage that is induced by MPTP via the *AKT*, *ERK*, and JNK signaling pathways.

In conclusion, the present study showed for the first time that Hepad possesses anti-oxidant activity and that it can exert neuroprotective effects against MPTP-induced oxidative stress injury. Specifically, our study demonstrated that Hepad protected against MPTP-induced injury by reducing the expression of inflammatory regulators (*TNF*-α, *IL-6*, *COX-2,* Mac-1, and *p*-*IκB*-α) and apoptosis-associated proteins (*p53*, *Bax*, *caspase-3*, and *PARP-1*), as well as by increasing the levels of anti-oxidant enzymes (*SOD*, *GST*, and *NOX-4*) via the *p*-*AKT* and MAPK (*p*-*ERK* and *p*-*JNK*) signaling pathways. Therefore, our findings suggest that Hepad is a potential therapeutic supplement for the mitigation and treatment of MPTP-induced cell and brain injury.

## 4. Materials and Methods

### 4.1. Sample Preparation

Samples of H1 and H2 were provided by the Department of Oriental Medicine and Traditional and Biomedical Research Center of Daejeon University. H1 consisted of *Atractylodis Rhizoma*, *Cnidii Rhizoma*, *Paeonia japonica*, *Poria cocos Wolf*, *Uncariae Ramulus et Uncus*, and *Zizyphi Semen* at equal ratios; H2 (S5) consisted of *Paeonia japonica, Uncariae Ramulus et Uncus*, and *Machilus thunbergii* at equal ratios. The herbs were extracted in boiling water for 24 h, and the extracts were then collected and filtered. Subsequently, the filtrate was concentrated under reduced pressure at 50 °C.

### 4.2. Cell Culture

Human neuroblastoma cells (SH-SY5Y) (Korean Cell Bank, Seoul, Korea) were cultured in Dulbecco’s modified Eagle’s medium containing 10% fetal bovine serum (Hyclone, Logan, UT, USA) and 1% penicillin-streptomycin (GIBCO, Grand Island, NY, USA) in a humidified incubator with 5% CO_2_ at 37 °C.

### 4.3. Cell Viability

We cultured SH-SY5Y (2 × 10^6^) cells in a 96-well plate, and cell viability was determined using the MTT (Promega, Madison, WI, USA) assay, as described previously [54]. Briefly, the cells were treated with various concentrations (0, 0.5, 1, 2, 4 and 5 mM) of MPTP (Sigma Aldrich, St. Louis, MO, USA); some cells were then treated with various concentrations (200–700 μg/mL) of H1 or H2 for 24 h. The MTT solution was then added, followed by incubation at 37 °C for 4 h. The supernatant was removed, and the formed formazan crystals were dissolved in dimethyl sulfoxide. The absorbance of the resulting solution was measured at 540 nm using a UVM 340 microplate reader (Biochrom Asys, Cambridge, UK).

### 4.4. Animal Experiments

**Figure 7:**
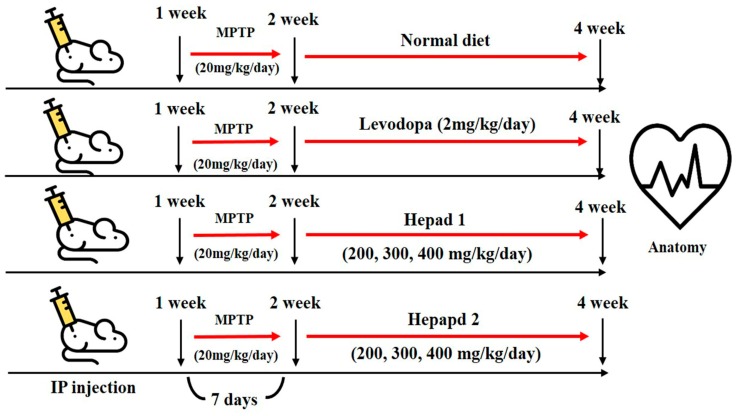
Flowchart depicting the timeline of the experimental procedures.

All experimental animals were approved by the Institutional Animal Care and Use Committee at Konkuk University (IACUC approval number, KU18045), Seoul, Korea. Seven-week-old male C57BL/6 mice were purchased from Orient Bio, Inc. (Seongnam-si, Korea) and housed in a temperature-controlled (21–22 °C) and light-controlled (12 h light/dark cycle) environment with 70% humidity; mice were given free access to water and rodent chow. After a 1-week adaptation period, the mice were randomly divided into nine groups (n = 8 per group) for MPTP, H1, and H2 studies, as follows: (1) normal (sham) group, (2) MPTP-intoxicated group, (3) positive group (MPTP + levodopa [2 mg/kg] [Sigma Aldrich]), (4) MPTP + H1 (200 mg/kg), (5) MPTP + H1 (300 mg/kg), (6) MPTP + H1 (400 mg/kg), (7) MPTP + H2 (200 mg/kg), (8) MPTP + H2 (300 mg/kg), and (9) MPTP + H2 (400 mg/kg). The mice in the sham group (group 1) had a normal diet for 28 days, and the mice in the MPTP-intoxicated group (group 2) were intraperitoneally injected with MPTP (20 mg/kg/day) for 28 days. In groups 3–9, MPTP (20 mg/kg/day) was administered for 7 days; then, levodopa (2 mg/kg/day), H1 (200, 300, and 400 mg/kg/day), or H2 (200, 300, and 400 mg/kg/day) were orally administered to the mice for 21 days, according to the group designations listed above. After 28 days, the mice were sacrificed by decapitation following a 16-h fast (Figure 7). The brains were dissected and analyzed by histological and Western blotting analyses. The brain tissues were stored at −70 °C in a deep freezer (ILSINTECH, Daejeon, Korea) until analysis.

### 4.5. Histological Analysis

Brain specimens (4 μm) were fixed in 10% formaldehyde for 24 h and were embedded in paraffin. The sections were stained with H&E for routine histopathological examination and were assessed using a light microscope (Eclipse TE 200; Nikon, Tokyo, Japan) at 100× magnification.

### 4.6. Immunohistochemical Staining

Brain sections (4-µm thick) were deparaffinized in a xylene-alcohol series for 5 min each, with subsequent recovery of anti-genic sites on steam fluent (pot value) for 30 min. The slides were washed twice for 5 min each with 1× phosphate-buffered saline, and then immersed in 0.3% hydrogen peroxide for 30 min at 25 °C to block endogenous peroxidase activity. The sections were incubated with 10% goat serum (for polyclonal antibodies) for 30 min, and then incubated at 4 °C with a primary antibody against TH (Abcam, Cambridge, UK) for 24 h. Subsequently, all samples were incubated with biotinylated goat anti-rabbit immunoglobulin G (H+L) horseradish peroxidase-conjugated antibodies (Zymax, San Francisco, CA, USA). The sections were incubated with 3,3-diaminobenzidine (Vector Laboratories, Inc., Burlingame, CA, USA) for 10 min at 37 °C. Finally, the tissue sections were counterstained with hematoxylin for 2 min, dehydrated with an alcohol-xylene series, and mounted with coverslips using Permount mounting medium (Thermo Fisher Scientific, Waltham, MA, USA). The specimens were examined using a Nikon Eclipse TS100 microscope (Nikon) at 200× magnification, and the microscopy images were analyzed using the OptiView image analysis software (Korea Lab Tech, Seongnam-si, Korea). Immunopositive neurons were counted manually.

### 4.7. Western Blotting Analysis

Mouse brains were lysed in radioimmunoprecipitation assay lysis buffer containing protease inhibitor (Roche, Mannheim, Germany) and centrifuged at 10,000× *g* for 30 min at 4 °C. Total protein levels were determined using a Bio-Rad protein kit (Bio-Rad, Hercules, CA, USA). The proteins were separated by electrophoresis in a 10–15% sodium dodecyl sulfate-polyacrylamide gel and transferred onto the Immobilon-P transfer membrane (EMD Millipore Co., Bedford, MA, USA). The membrane was blocked with 5% bovine serum albumin (Sigma Aldrich). Subsequently, the membrane was incubated at 4 °C for 24 h with primary antibodies against one of the following proteins: *β-actin* (Cell Signaling Technology, Beverly, MA, USA), *TNF*-α (Abcam), *IL-6* (Santa Cruz Biotechnology, Santa Cruz, CA, USA), *Mac-1* (Bio-Rad), *iNOS* (Abcam), *COX-2* (Abcam), *Bcl-2* (Abcam), *GST* (Cell Signaling Technology), *SOD-1* (Santa Cruz Biotechnology), *NOX-4* (Novusbio, Littleton, CO, USA), *Bax* (Cell Signaling Technology), *caspase-3* (Abcam), *PARP-1* (Abcam), *p*-*AKT* (Cell Signaling Technology), *p*-*ERK* (Cell Signaling Technology), and *p*-*JNK* (Cell Signaling Technology). The membranes were subsequently incubated with goat anti-rabbit immunoglobulin G (H+L) horseradish peroxidase-conjugated secondary antibody (Zymax). Protein bands were detected using a chemiluminescence method (Thermo Fisher Scientific) by a C-DiGit Blot Scanner (Li-COR, Lincoln, NE, USA), and their densities were quantified using ImageJ (NIH, Rockville, MD, USA). All data were normalized to the *β-actin* values.

### 4.8. Statistical Analyses

All statistical analyses were performed using SPSS version 18.0 (IBM, Chicago, IL, USA). One-way analyses of variance with Duncan’s post hoc tests were used to identify differences in the mean values between experimental groups. Data for each test are presented as mean ± standard deviations. Statistical significance was set at *p* < 0.05.
